# Study of wrist joint motion with dynamic radiography

**DOI:** 10.1186/1753-6561-9-S3-A107

**Published:** 2015-05-19

**Authors:** Kaoru Tada

**Affiliations:** 1 Department of Orthopaedic Surgery, Kanazawa University, 920-1302, Kanazawa, Japan

## 

Dynamic radiography had been used for motion analysis of the wrist joint since 1980s. But only limited images could be obtained with high exposure doses in conventional equipment. In recent years, advance equipment for dynamic radiography was developed, and thus, various images could be obtained with low exposure doses and without distortion. In this session, I would like to talk about our studyof the radiocarpal and midcarpal joint motion with dynamic radiography.

## Study 1: “Wrist rhythm” during the wrist joint exercise

Our hypothesis is that there is “wrist rhythm” during wrist exercise eg. scapulohumeral rhythm. Therefore we evaluated the ratio of motion of the radiocarpal and midcarpal joints during wrist joint exercise by dynamic radiography. The subjects were 40 wrists of 20 healthy men without past trauma or disorder around the wrists. Dynamic radiography was conducted while creating active wrist joint exercise. In this study, the wrist angle is defined as the angle between the line of the dorsal aspect of the radius (line A) and the line of the dorsal aspect of the 3rd metacarpal bone (line B). The radiocarpal angle is defined as the angle between line A and the line perpendicular to the distal articulation of the lunate (line C). The midcarpal angle is defined as the angle between lines B and C. An average curve with the horizontal axis as the wrist angle, and the vertical axes as the radiocarpal and midcarpal angles, respectively, was created. When the regression line was calculated using the least-squares method from the average curve, it became clear that the ratio of the radiocarpal and midcarpal joints motions was approximately 1:4 in palmar flexion, and 2:1 in dorsiflexion. We would like to propose these results as wrist rhythm. We expect wrist rhythm will serve as an aid for understanding the complicated motion of the wrist joint.

## Study 2: Effects of traction on the wrist joint exercise

We perform passive range of motion (ROM) exercise of the wrist joint under traction in patients with disorders of the wrist joint because exercise under traction is considered to reduce the load on the joint surface and stretch the soft tissues around the joint. However, there were no reports on the dynamic effect of traction on the wrist joint in a living body, and the effect of ROM exercise of the wrist joint under traction was still unclear. We described the dynamic effects of traction on the radiocarpal and midcarpal joints in a living body to seek the possibility of range of motion exercise of the wrist joint under traction as a new method of exercise. The subjects were 20 wrists of 20 healthy menwithout past trauma or disorder around the wrists. Dynamic radiography was conducted while creating passive wrist joint exercise by using manual traction and machinery traction with measurement device that enabled the control of traction power and the direction of traction during exercise for quantitative analysis of the traction effect. The change in the percent contribution of the radiocarpal and midcarpal joints with or without traction were calculated. In the results, the percent contribution of the radiocarpal joint increased and that of midcarpal joint decreased with the addition of traction during both palmar flexion and dorsiflexion. The results with manual traction and machinery traction were almost same. Range of motion exercise with traction has the potential to be applied to an advanced rehabilitation program targeting the radiocarpal joint under specific pathological conditions.

